# Sirt1 Deletion Leads to Enhanced Inflammation and Aggravates Endotoxin-Induced Acute Kidney Injury

**DOI:** 10.1371/journal.pone.0098909

**Published:** 2014-06-04

**Authors:** Rong Gao, Jiao Chen, Yuxin Hu, Zhenyu Li, Shuxia Wang, Sreerama Shetty, Jian Fu

**Affiliations:** 1 The Second Hospital of Jilin University, Changchun, Jilin, China; 2 Center for Research on Environmental Disease, College of Medicine, University of Kentucky, Lexington, Kentucky, United States of America; 3 Graduate Center for Toxicology, College of Medicine, University of Kentucky, Lexington, Kentucky, United States of America; 4 Division of Cardiovascular Medicine, College of Medicine, University of Kentucky, Lexington, Kentucky, United States of America; 5 Graduate Center for Nutritional Sciences, College of Medicine, University of Kentucky, Lexington, Kentucky, United States of America; 6 Center for Biomedical Research, University of Texas Health Science Center at Tyler, Tyler, Texas, United States of America; University of North Dakota, United States of America

## Abstract

Bacterial endotoxin has been known to induce excessive inflammatory responses and acute kidney injury. In the present study, we used a mouse model of endotoxemia to investigate the role of Sirt1 in inflammatory kidney injury. We examined molecular and cellular responses in inducible Sirt1 knockout (Sirt1^−/−^) mice and wild type littermates (Sirt1^+/+^) in lipopolysaccharide (LPS)-induced kidney injury. Our studies demonstrated that Sirt1 deletion caused aggravated kidney injury, which was associated with increased inflammatory responses including elevated pro-inflammatory cytokine production, and increased ICAM-1 and VCAM-1 expression. Inflammatory signaling such as STAT3/ERK phosphorylation and NF-κB activation was markedly elevated in kidney tissues of Sirt1 knockout mice after LPS challenge. The results indicate that Sirt1 is protective against LPS-induced acute kidney injury by suppressing kidney inflammation and down-regulating inflammatory signaling.

## Introduction

Sepsis arises mostly from bacterial infection which causes multiple organ failure due to excessive systemic inflammation [1], [2]. Kidney functions as a natural filter of blood and serves as the first line of defense in our body [3], [4]. Unfortunately, it also becomes a direct target of inflammatory injury [5]. Sepsis-induced acute kidney injury (AKI) is very common in the elderly and associated with high mortality [6]–[8]. To date, there has been no effective treatment for this devastating disease [9], [10]. Lipopolysaccharide (LPS) challenge is one of the most accepted animal models to explore the underlying mechanisms and potential treatment in sepsis-induced kidney injury [11].

Renal function is significantly compromised during sepsis as indicated by increased blood urea nitrogen (BUN) and urine Kidney injury molecule-1 (KIM-1) levels [12]–[14]. Sepsis also causes renal tubular damage and inflammation as shown by histological analysis [15]. Kidney inflammation is associated with increased production of pro-inflammatory mediators [16], and up-regulation of adhesion molecules such as Intercellular adhesion molecule-1 (ICAM-1) and vascular cell adhesion molecule-1 (VCAM-1) [17]. Those early inflammatory responses induce leukocyte infiltration during kidney injury [18], which may lead to further damage. Increased cytokine production is a hallmark in many inflammatory diseases including kidney injury [19], [20]. Cytokines can induce pro-inflammatory signaling such as activatation of signal transducer and activator of transcription 3 (STAT3) [21], and modulate inflammatory responses through ERK/MAPK cascade [22].

Sirt1, a member of the Sirtuin family [23], is a deacetylase that has been reported to modulate the function of a wide variety of proteins such as NF-κB and p53, through deacetylation of lysine residues [24]. There have been increasing studies suggesting that Sirt1 plays an important role in inflammation, apoptosis, stress resistance, metabolism, differentiation, and aging [8], [25]–[31]. In the present study, we investigated the role of Sirt1 in LPS-induced acute kidney injury by inducible deletion of Sirt1 in mice. Our studies demonstrate that Sirt1 knockout mice are highly susceptible to LPS-induced inflammatory kidney injury.

## Materials and Methods

### Reagents

Tamoxifen and lipopolysaccharide (Escherichia coli serotype 0111:B4) were purchased from Sigma-Aldrich (St. Louis, MO, USA). TNF-α and IL-6 ELISA kits were obtained from Biolegend (San Diego, CA, USA). Blood urea nitrogen (BUN) assay kit was obtained from Arbor Assays (Ann Arbor, Michigan, USA). Kidney injury molecule-1 (KIM-1) assay kit was purchased from R&D (Minneapolis, MN, USA). Anti-Mouse Ly-6G (Gr-1)-FITC was purchased from eBioscience (San Diego, CA, USA). Goat anti-mouse ICAM-1 and VCAM-1 antibodies were purchased from Santa Cruz Biotechnology (Dallas, Texas, USA). Phospho-STAT3 (Thr705), Stat3 (124H6), phospho-p44/42MAPK (ERK1/2) (Thr202/Tyr204), p44/42MAPK (ERK1/2), phosphorylated IκBα, IκBα, and β-actin antibodies were obtained from Cell Signal Technology (Boston, MA, USA).

### Animal model of acute kidney injury

Mice were housed in cages with free access to food and water in a temperature controlled room with a 12-hour dark/light cycle. All experiments and animal care procedures were approved by the Institutional Animal Care and Use Committee of the University of Texas Health Science Center at Tyler. The generation of inducible Sirt1 knockout mice was described previously [32], [33]. Six to seven weeks after the birth, mice were given tamoxifen (100 mg/Kg body weight in corn oil) by intraperitoneal (I.P.) injection daily for 5 days to induce nuclear translocation of Cre recombinase as described previously [33]. Fourteen to fifteen weeks after Sirt1 deletion, age-matched male Sirt1^−/−^ and Sirt1^+/+^ littermates were used in the studies. Endotoxemia was induced by I.P. injection of 5 mg/kg LPS dissolved in phosphate buffered saline (PBS), control mice were injected with PBS. Experiments were terminated 6 or 24 h after LPS challenge.

### Renal function assay and histology analysis

Blood and urine samples were obtained from mice 24 h after LPS challenge, serum BUN and urine KIM-1 levels were examined as markers of renal dysfunction. Paraffin-embedded sections of mouse kidney tissues were stained with hematoxylin and eosin for assessment of renal tubular injury. The histological samples were scored by lab personnel blinded to the samples. The magnitude of tubular injury including tubular dilatation, flattening and vacuolization was scored into five levels (0, none; 1, 0–25%; 2, 25 to 50%; 3, 50 to 75%; and 4, >75%) on the basis of the percentage of affected tubules in a high-power field under light microscope.

### ELISA, Immunofluorescence, and Immunoblotting assays

Serum IL-6 and TNF-α levels were determined by ELISA kits (Biolegend). Blood samples were collected 6 and 24 h after LPS challenge. Kidneys samples were obtained 6 h or 24 h after LPS exposure. Immunofluorescence and immunoblotting assays were conducted as described previously [34].

### Statistical analysis

Data were analyzed by two-way ANOVA followed by Bonferroni's multiple comparisons tests and expressed as mean ± SEM. Statistical significance was assigned to P values less than 0.05.

## Results

### Sirt1 deletion leads to aggravated renal dysfunction after LPS challenge

**Figure 1:**
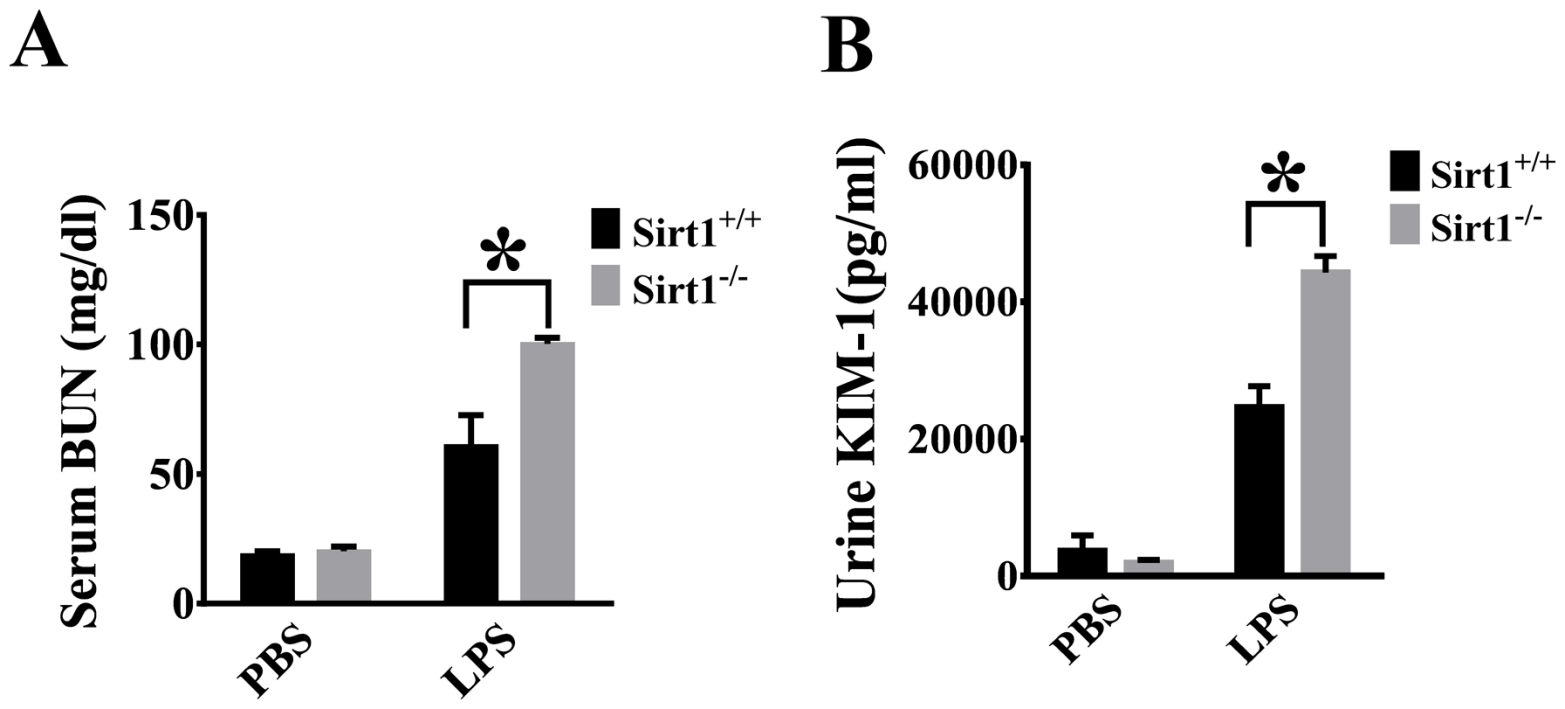
Aggravated renal dysfunction in Sirt1 knockout mouse after LPS challenge. Sirt1^−/−^ mice and Sirt1^+/+^ littermates were divided into four groups (Sirt1^+/+^/PBS, Sirt1^−/−^/PBS, Sirt1^+/+^/LPS, Sirt1^−/−^/LPS). Serum BUN (A) and KIM-1(B) Levels were measured 24 h after LPS challenge n≥4 mice/group. * P<0.05 versus LPS/Sirt1^+/+^ group.

Serum BUN and urine KIM-1 levels were used as markers of kidney function [14], [35]. BUN and KIM-1 levels were increased after LPS challenge, and significantly higher in Sirt1 knockout mice than the wild type littermates (Figure 1 A, B), indicating exacerbated renal dysfunction in Sirt1 knockout mice.

**Figure 2:**
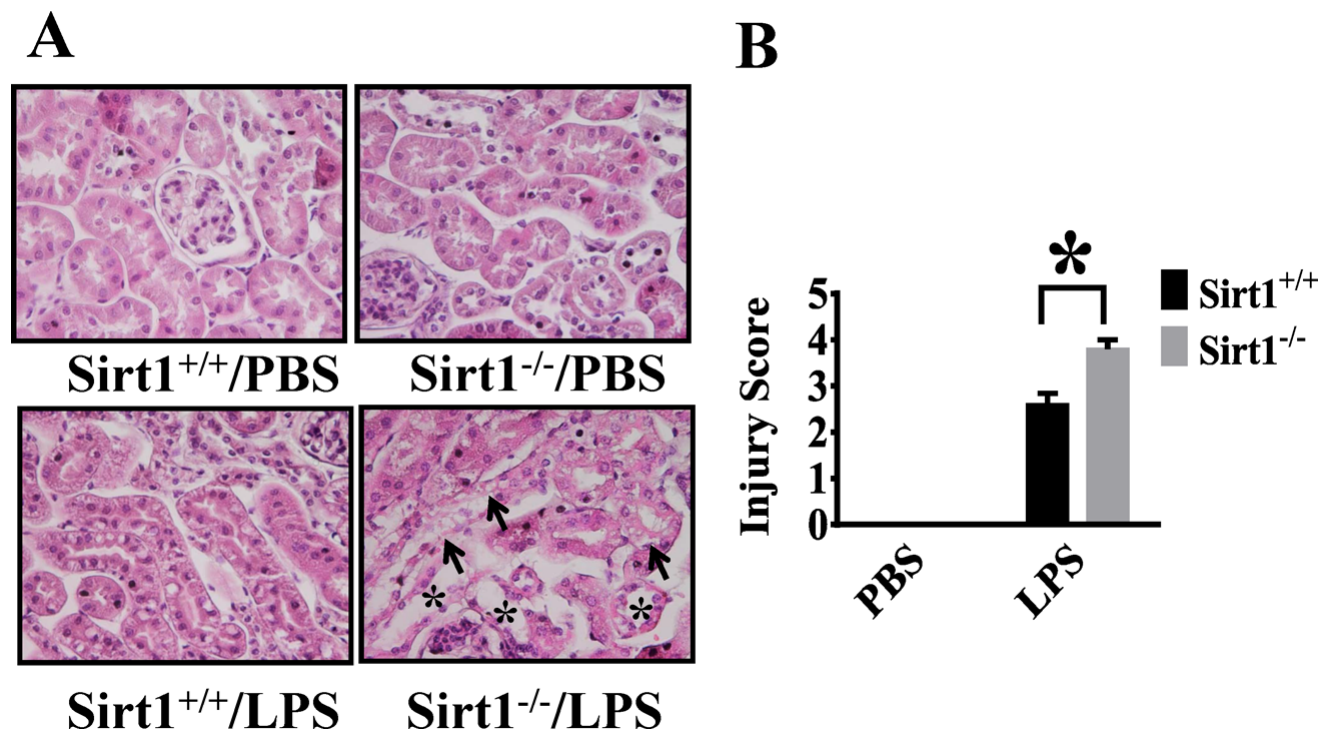
Aggravated renal tubular injury in Sirt1 knockout mice after LPS challenge. Kidney tissues were harvested 24(n = 5 mice/group). The collected kidneys were stained with H&E staining. (A) Histological examination shows increased tubular injury in kidney cortex of Sirt1^−/−^ mice after LPS challenge, including tubular dilatation, flattening (*) and vacuolization (arrows). (B) Quantitative evaluation of morphological tubular damage 24 h after LPS challenge. *P<0.05 versus Sirt1^+/+^/LPS group.

LPS-induced kidney injury was examined in the tubules of the kidney cortex. The mice without LPS challenge showed normal and healthy kidney histology. We detected severe structural damage in the kidneys of Sirt1^−/−^ mice (Figure 2). Tubular injury including tubular dilatation, flattening and renal tubular cell vacuolization were markedly increased in Sirt1 ^−/−^ mice when compared with Sirt1^+/+^ littermates. Tubular injury scores indicate that kidney injury was significantly aggravated in Sirt1 knockout mice.

### Sirt1 deletion leads to increased production of pro-inflammatory cytokines after LPS challenge

**Figure 3:**
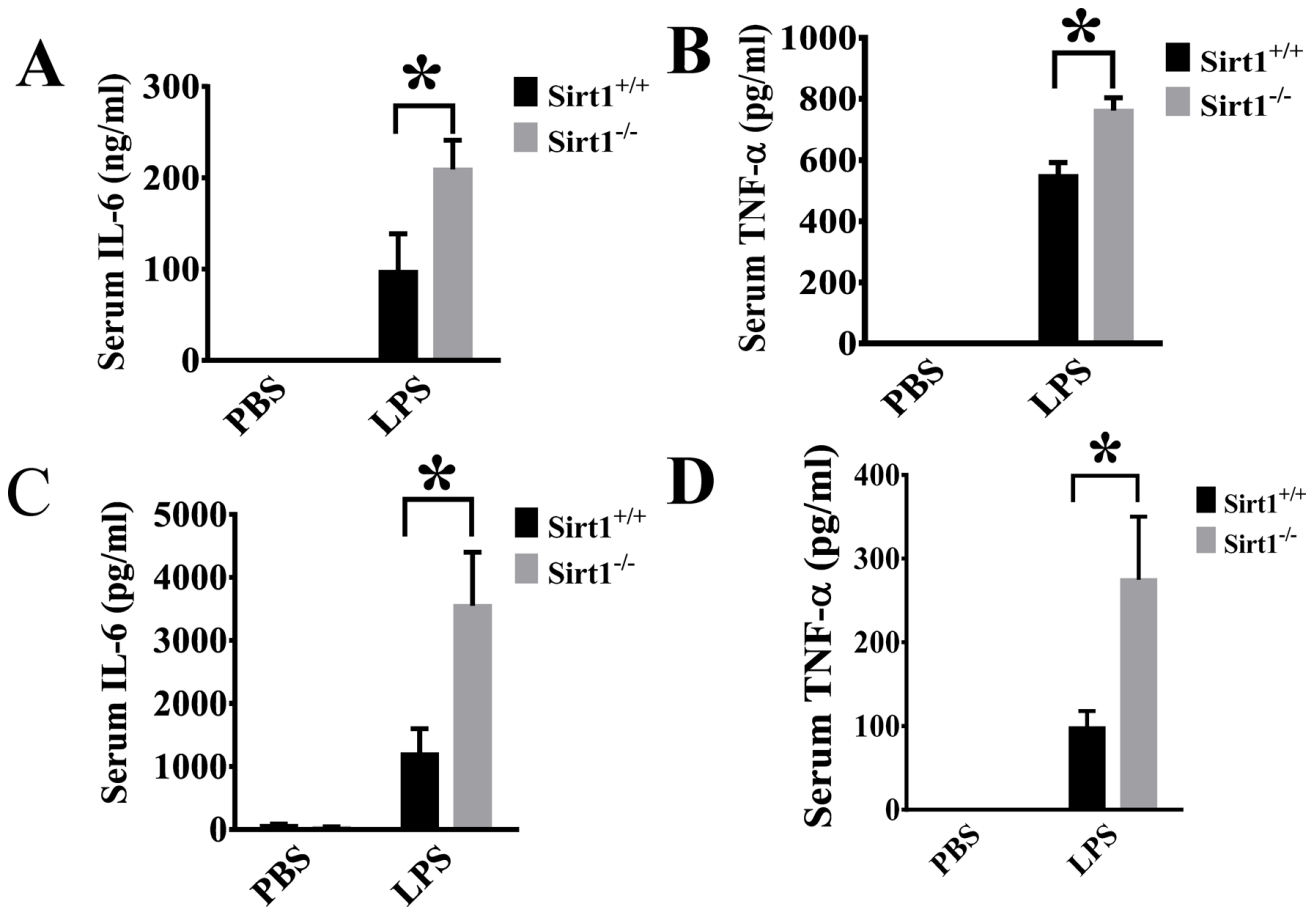
Sirt1 deletion causes significant increase of pro-inflammatory cytokine production after LPS challenge. Serum IL-6 and TNF-alpha levels were measured by ELISA 6 h (A, B) or 24 h (C, D) after LPS challenge (n≥4 mice/group). *P<0.05 versus Sirt1^+/+^/LPS group.

The effects of Sirt1 deletion on systemic inflammatory responses were determined by examining serum IL-6 and TNF-α levels. We observed that LPS-induced IL-6 and TNF-α production were significantly increased in Sirt1^−/−^ mice when compared with Sirt1^+/+^ littermates after LPS exposure (Figure 3), suggesting that Sirt1 modulates systemic production of pro-inflammatory cytokines.

### Sirt1 deletion leads to increased neutrophil infiltration in the kidney after LPS challenge

**Figure 4:**
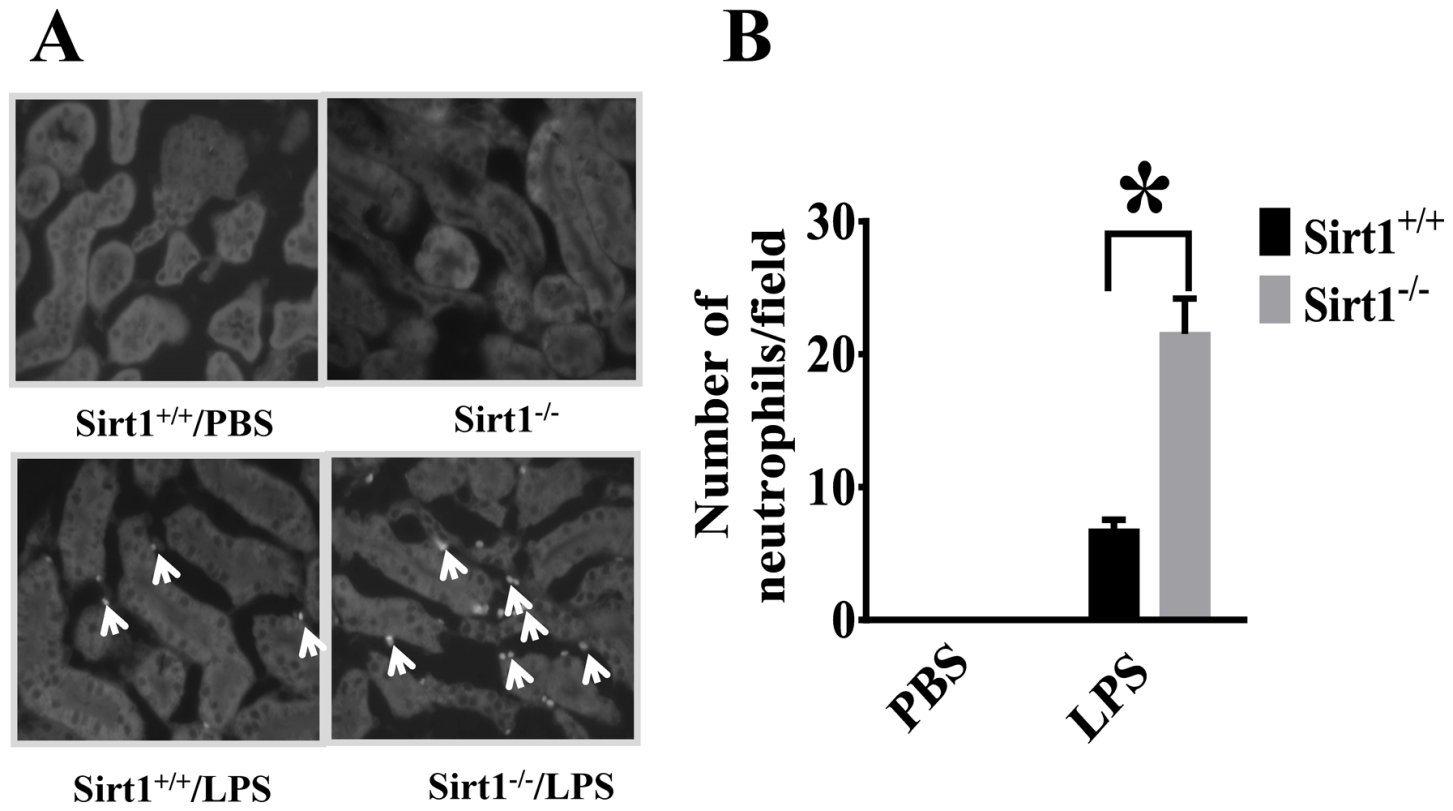
Increased neutrophil infiltration in Sirt1 knockout mice after LPS challenge. Twenty four hours after LPS challenge, kidney tissues from Sirt1^−/−^ mice and sirt1^+/+^ littermates were collected (n = 5 mice/group). Cryosections were prepared. (**A**) Gr-1 was used as a specific marker for neutrophil staining. Neutrophil (arrow) infiltration was detected. (B) The number of neutrophils was counted. *P<0.05 versus Sirt1^+/+^/LPS group.

To further assess the effects of Sirt1 deletion on LPS-induced kidney inflammation, neutrophil infiltration into the kidney was examined using neutrophil-specific Gr-1 antibody. No neutrophil infiltration was detected in the control group. LPS challenge led to increased neutrophil infiltration in the kidney, which was much more severe in Sirt1 knockout mice (Figure 4 A, B).

### Sirt1 deletion leads to increased ICAM-1/VCAM-1 expression in the kidney after LPS challenge

**Figure 5:**
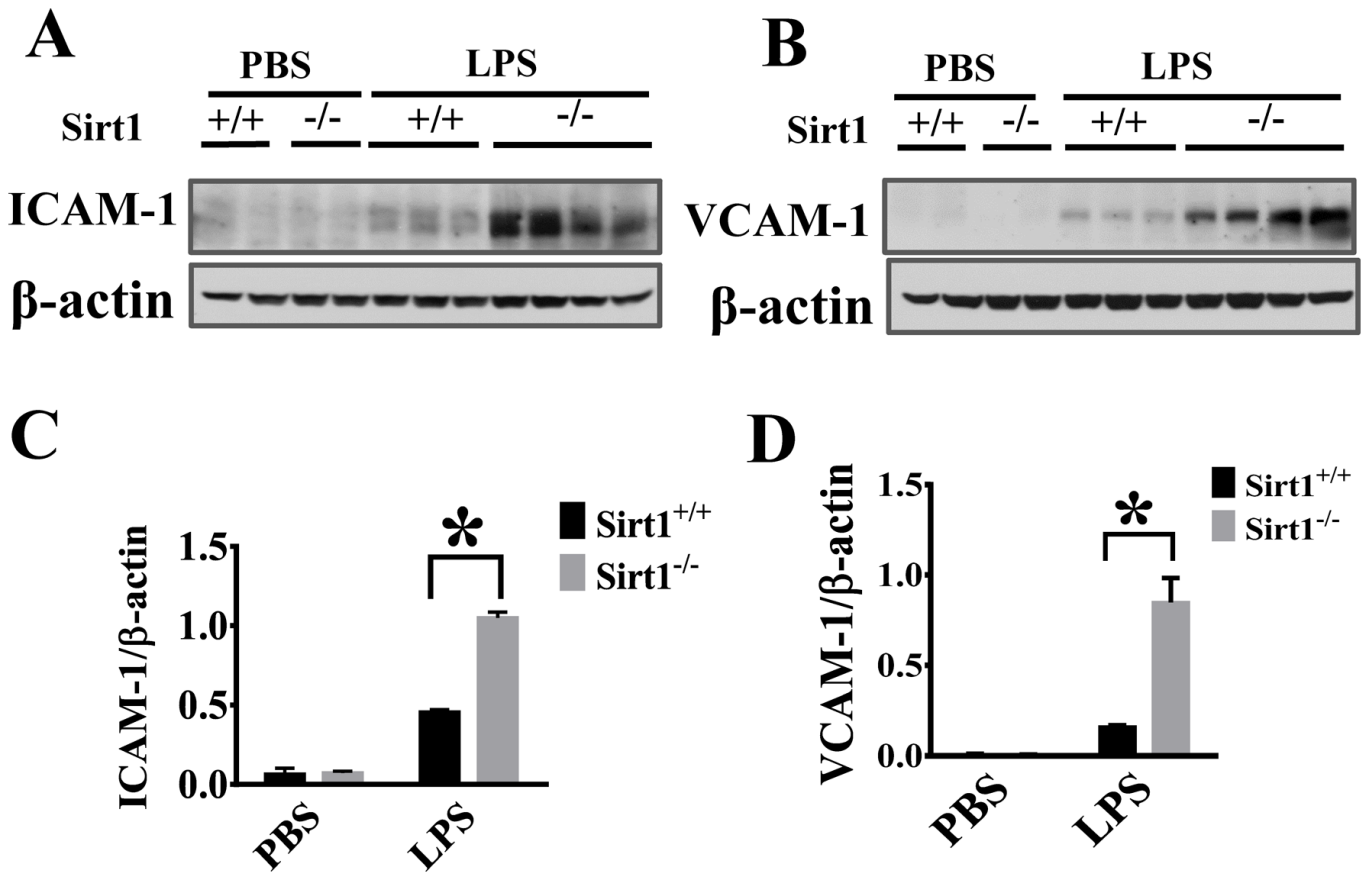
Sirt1 deletion enhances LPS-induced ICAM-1/VCAM-1 expression in the kidney. ICAM-1 and VCAM-1expression were assayed 24 h after LPS challenge (n≥4 mice/group). (A, B) Representative blots showing ICAM-1 and VCAM-1 expression in the kidney. (C, D) Densitometry analysis. *P<0.05 versus Sirt1^+/+^/LPS group.

Adhesion molecules on vascular endothelial cells are major determinants of vascular inflammation [36]. We examined the effects of Sirt1 deletion on LPS-induced VCAM-1 and ICAM-1 expression in kidney tissues. Immunoblotting assays showed that Sirt1 knockout mice exhibited significantly higher ICAM-1/VCAM-1 expression after LPS challenge than Sirt1^+/+^ littermates (Figure 5 A–D).

### Sirt1 deletion leads to enhanced inflammatory signaling in the kidney after LPS challenge

**Figure 6:**
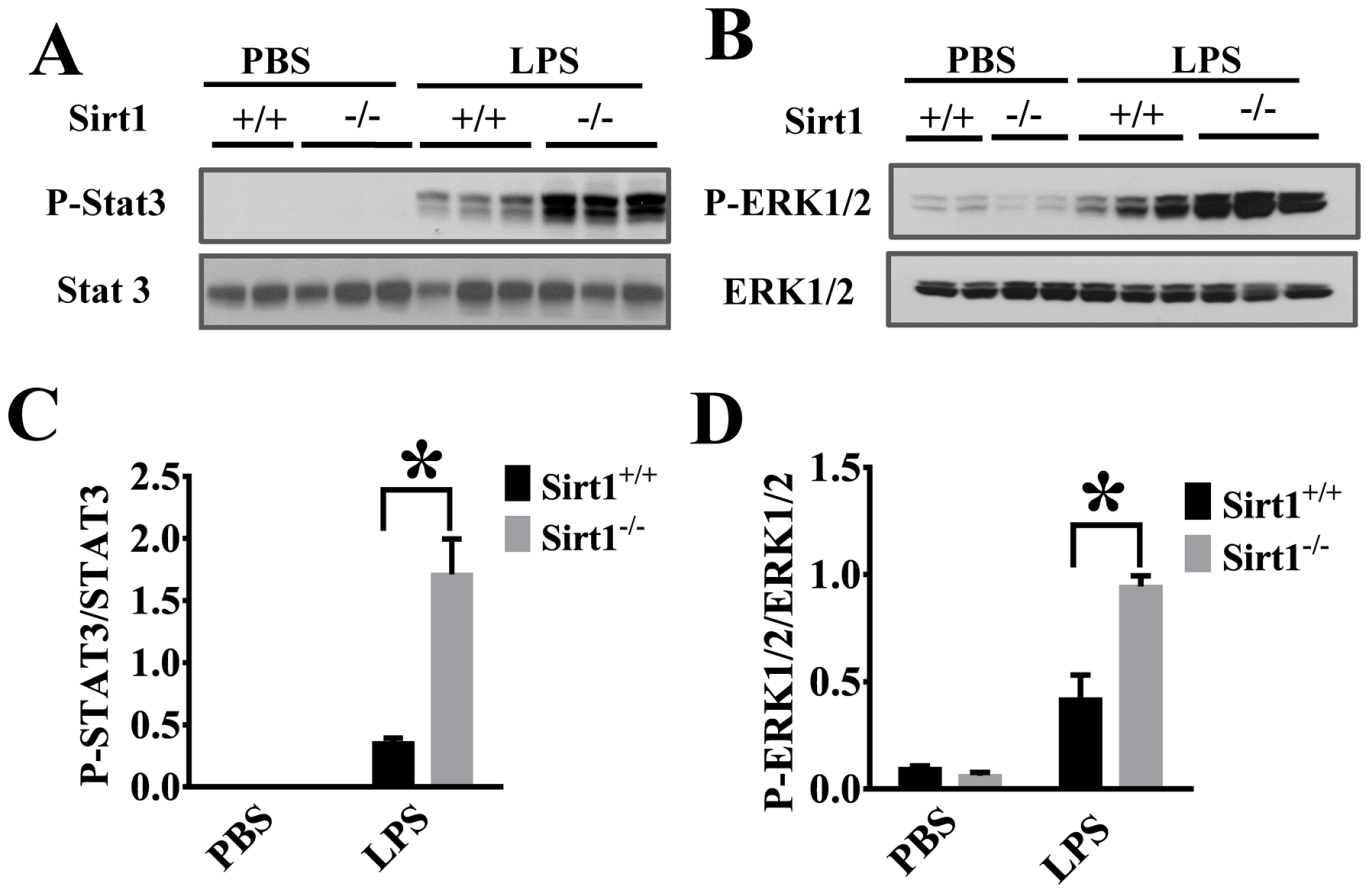
Sirt1 deletion enhances LPS-induced Stat3 and ERK1/2 activation in the kidney. Kidney tissues were harvested 6(n≥7 mice/group). Samples were subjected to immunoblotting assay. STAT3 (A) and ERK1/2 (B) phosphorylation were examined in the kidney tissues by immunoblotting assay. (C, D) Densitometry analysis. *P<0.05 versus Sirt1^+/+^/LPS group.

**Figure 7:**
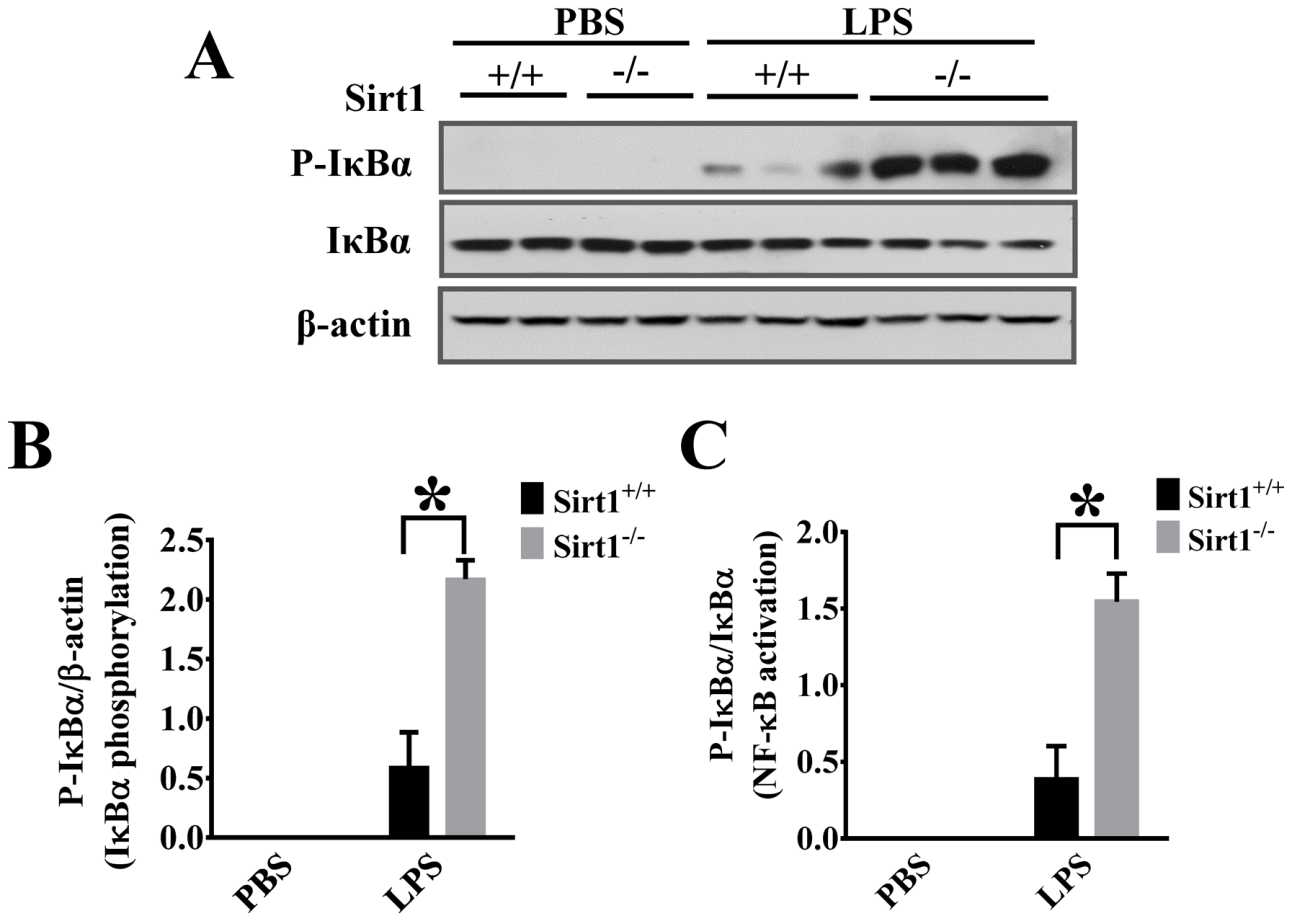
Sirt1 deletion enhances LPS-induced NF-κB activation. Kidney tissues were harvested 6(n≥7 mice/group). Kidney samples were then subjected to immunoblotting assay. IκBα phosphorylation and IκBα level were examined. (A) Representative blots of IκBα phosphorylation and total IκBα. (B, C) Densitometry analysis. *P<0.05 versus Sirt1^+/+^/LPS group.

We then conducted experiments to investigate the mechanisms of sirt1 regulation of the kidney inflammation. Inflammatory signaling including Stat3 pathway has been reported to play an important role in the development of kidney injury [37]. Our results indicate that STAT3 phosphorylation was increased in Sirt1^−/−^ mice when compared with Sirt1^+/+^ littermates (Figure 6A, C). To confirm Sirt1 regulation of inflammatory signaling, we also examined ERK phosphorylation in the kidney after LPS challenge. The results showed that Sirt1 deletion caused significant increases in ERK1/2 phosphorylation (Figure 6B, D). Furthermore, Sirt1 deletion led to increased NF-κB activation in kidney tissues after LPS challenge as demonstrated by higher IκBα phosphorylation and degradation (Figure 7), suggesting that Sirt1 down-regulation of inflammatory signaling could be one of the mechanisms contributing to its protection against kidney inflammation.

## Discussion

Sepsis remains a devastating disease without cure. While many studies have been done to explore the treatment for the disease [38]–[42], the mechanisms of septic shock and associated multi-organ failure are yet to be determined [43], [44]. Endotoxemia is responsible for sepsis-induced excessive inflammatory injury including acute kidney injury in many clinic settings [45]. The Kidney, as a maintainer of internal environment homeostasis [3], [4], is vulnerable to tissue injury caused by toxins and pro-inflammatory mediators [6], and studies are needed to identify protective mechanisms against sepsis-induced kidney injury.

Altered Sirt1 expression and function have been associated with many pathological changes [46]. We reported previously that Sirt1 plays an important role in regulating lung inflammation and coagulation responses [33]. Interestingly, Sirt1 expression is reduced during aging and in some pre-existing inflammatory diseases such as COPD and alcoholic fatty liver disease [47], [48], and the elderly are also highly susceptible to inflammatory disorders including sepsis [23], [47], [48].

The progression of sepsis is likely associated with two phases. A systemic inflammatory response syndrome (SIRS) phase and a compensatory anti-inflammatory response syndrome (CARS) phase. However, not all patients who suffer from SIRS develop into severe sepsis [49]. Tight control of the balance between the two phases could be an important means of suppressing excessive inflammation [50]. Our studies demonstrate that Sirt1 deletion leads to aggravated inflammatory kidney injury. Given the reduced Sirt1 expression during aging [51], our results may provide an insight on why the elderly are more susceptible to sepsis-associated kidney injury. Therefore, Sirt1 could be considered as a potential target to treat inflammatory kidney injury in the aged population.

Pro-inflammatory cytokines such as TNF-α and IL-6 have been known to play a critical role in sepsis-induced inflammatory injury [19], [43]. Our results showed that Sirt1 deletion led to increased LPS-induced IL-6 and TNF-α production, suggesting that Sirt1 acts to suppress inflammatory responses during sepsis. The cytokines is also known to play an important role in kidney injury [37]. Inflammatory signaling such as STAT-3 activation promotes kidney inflammatory responses [52]. In our studies, we showed that Sirt1 deletion led to enhanced pro-inflammatory signaling as demonstrated by increased STAT and ERK phosphorylation. Interestingly, Sirt1 expression in the kidney is higher in young mice and decreased during aging [53]. Even though it will be intriguing to link uncontrolled inflammatory responses in the elderly during sepsis to the reduced Sirt1 expression, more studies are needed to elucidate the role of Sirt1 in aging-related kidney injury.

Excessive neutrophil infiltration often leads to inflammatory tissue injury including acute kidney injury [18]. Leukocyte migration to the injured sites could help to remove dead cells and promote the repair process [54], however, excessive neutrophil accumulation can result in tissue damage [35]. Neutrophil infiltration across the vasculature is a multistep process which requires neutrophil/endothelial interactions through adhesion molecules ICAM-1 and VCAM-1 [36]. Our results demonstrated that Sirt1 deletion led to increased ICAM-1/VCAM-1 expression and neutrophil infiltration in the kidney after LPS challenge, suggesting that Sirt1 modulates some key factors needed for sustained inflammatory responses. Sirt1, a member of Class III Histone Deacetylases [55], modulates transcriptional activities of associated transcriptional factors, cofactors, and histones by controlling their acetylation [56], [57]. NF-κB pathway is a central signaling node in inflammatory cytokine production and activation [55]. NF-κB, a master transcription regulator which also controls ICAM-1/VCAM-1 expression [58], is an endogenous substrate of Sirt1 [24]. Deacetylation of NF-κB inhibits its activity and has been linked to the anti-inflammatory function of Sirt1 [59]. Our data indicate that Sirt1 modulates NF-κB pathway in the kidney including IκBα phosphorylation and degradation.

In summary, Sirt1 plays a protective role against inflammatory kidney injury in endotoxemia. Sirt1 exerts its function likely through multiple pathways such as suppressing STAT3, ERK1/2, and NF-κB activation. Our studies indicate that Sirt1 is a potential therapeutic target to treat sepsis-induced kidney injury.

## References

[1] Zhang D, Li Y, Liu Y, Xiang X, Dong Z (2013) Paclitaxel ameliorates lipopolysaccharide-induced kidney injury by binding myeloid differentiation protein-2 to block Toll-like receptor 4-mediated nuclear factor-kappaB activation and cytokine production. J Pharmacol Exp Ther 345: 69–75. 23318472 10.1124/jpet.112.202481 PMC3608443

[2] Jeong SJ, Han SH, Kim CO, Choi JY, Kim JM (2013) Anti-vascular endothelial growth factor antibody attenuates inflammation and decreases mortality in an experimental model of severe sepsis. Crit Care 17: R97. 23710641 10.1186/cc12742 PMC4056034

[3] Adachi M, Kitamura K, Tomita K (2005) [Regulation of sodium and water balance by the kidney]. Nihon Rinsho 63: 45–50. 15675316

[4] Horky K (1967) [Role of the renin-angiotensin system in homeostasis of the internal environment]. Cas Lek Cesk 106: 105–113. 4297563

[5] Coldewey SM, Khan AI, Kapoor A, Collino M, Rogazzo M, et al.. (2013) Erythropoietin attenuates acute kidney dysfunction in murine experimental sepsis by activation of the beta-common receptor. Kidney Int. 10.1038/ki.2013.118 23594675

[6] Suh SH, Kim CS, Choi JS, Bae EH, Ma SK, et al (2013) Acute kidney injury in patients with sepsis and septic shock: risk factors and clinical outcomes. Yonsei Med J 54: 965–972. 23709433 10.3349/ymj.2013.54.4.965 PMC3663224

[7] Maddens B, Vandendriessche B, Demon D, Vanholder R, Chiers K, et al (2012) Severity of sepsis-induced acute kidney injury in a novel mouse model is age dependent. Crit Care Med 40: 2638–2646. 22743777 10.1097/CCM.0b013e3182591ebe

[8] Lim JH, Kim EN, Kim MY, Chung S, Shin SJ, et al (2012) Age-associated molecular changes in the kidney in aged mice. Oxid Med Cell Longev 2012: 171383. 23326623 10.1155/2012/171383 PMC3544311

[9] Nguyen HB, Rivers EP, Abrahamian FM, Moran GJ, Abraham E, et al (2006) Severe sepsis and septic shock: review of the literature and emergency department management guidelines. Ann Emerg Med 48: 28–54. 16781920 10.1016/j.annemergmed.2006.02.015

[10] Venkataraman R, Kellum JA (2013) Sepsis: update in the management. Adv Chronic Kidney Dis 20: 6–13. 23265591 10.1053/j.ackd.2012.10.013

[11] McCurdy TR, Patrick AL, Eltringham-Smith LJ, Bhakta V, Sheffield WP, et al.. (2013) Alpha-1 acid glycoprotein reduces hepatic leukocyte recruitment in murine models of either early endotoxemia or early sepsis. Microcirculation. 10.1111/micc.12081 23941548

[12] Miyaji T, Hu X, Yuen PS, Muramatsu Y, Iyer S, et al (2003) Ethyl pyruvate decreases sepsis-induced acute renal failure and multiple organ damage in aged mice. Kidney Int 64: 1620–1631. 14531793 10.1046/j.1523-1755.2003.00268.x

[13] Peng X, Xu H, Zhou Y, Wang B, Yan Y, et al (2013) Human umbilical cord mesenchymal stem cells attenuate cisplatin-induced acute and chronic renal injury. Exp Biol Med (Maywood) 238: 960–970. 23956354 10.1177/1477153513497176

[14] Sabbisetti VS, Ito K, Wang C, Yang L, Mefferd SC, et al (2013) Novel assays for detection of urinary KIM-1 in mouse models of kidney injury. Toxicol Sci 131: 13–25. 23019274 10.1093/toxsci/kfs268 PMC3621351

[15] Wan L, Bagshaw SM, Langenberg C, Saotome T, May C, et al (2008) Pathophysiology of septic acute kidney injury: what do we really know? Crit Care Med 36: S198–203. 18382194 10.1097/CCM.0b013e318168ccd5

[16] Danoff TM (1998) Chemokines in interstitial injury. Kidney Int 53: 1807–1808. 9607217 10.1046/j.1523-1755.1998.00920.x

[17] Kasprzak A, Surdacka A, Tomczak M, Konkol M (2013) Role of high endothelial postcapillary venules and selected adhesion molecules in periodontal diseases: a review. J Periodontal Res 48: 1–21. 22582923 10.1111/j.1600-0765.2012.01492.x

[18] Lampinen M, Sangfelt P, Taha Y, Carlson M (2008) Accumulation, activation, and survival of neutrophils in ulcerative colitis: regulation by locally produced factors in the colon and impact of steroid treatment. Int J Colorectal Dis 23: 939–946. 18594843 10.1007/s00384-008-0509-x

[19] Greenhill CJ, Rose-John S, Lissilaa R, Ferlin W, Ernst M, et al (2011) IL-6 trans-signaling modulates TLR4-dependent inflammatory responses via STAT3. J Immunol 186: 1199–1208. 21148800 10.4049/jimmunol.1002971

[20] Mihara M, Hashizume M, Yoshida H, Suzuki M, Shiina M (2012) IL-6/IL-6 receptor system and its role in physiological and pathological conditions. Clin Sci (Lond) 122: 143–159. 22029668 10.1042/CS20110340

[21] Tu B, Du L, Fan QM, Tang Z, Tang TT (2012) STAT3 activation by IL-6 from mesenchymal stem cells promotes the proliferation and metastasis of osteosarcoma. Cancer Lett 325: 80–88. 22743617 10.1016/j.canlet.2012.06.006

[22] Wang S, Wei Q, Dong G, Dong Z (2013) ERK-mediated suppression of cilia in cisplatin-induced tubular cell apoptosis and acute kidney injury. Biochim Biophys Acta 1832: 1582–1590. 23727409 10.1016/j.bbadis.2013.05.023 PMC3752396

[23] Houtkooper RH, Pirinen E, Auwerx J (2012) Sirtuins as regulators of metabolism and healthspan. Nat Rev Mol Cell Biol 13: 225–238. 22395773 10.1038/nrm3293 PMC4872805

[24] Hao CM, Haase VH (2010) Sirtuins and their relevance to the kidney. J Am Soc Nephrol 21: 1620–1627. 20595677 10.1681/ASN.2010010046 PMC4527176

[25] Chang HC, Guarente L (2013) SIRT1 Mediates Central Circadian Control in the SCN by a Mechanism that Decays with Aging. Cell 153: 1448–1460. 23791176 10.1016/j.cell.2013.05.027 PMC3748806

[26] Uribarri J, Cai W, Pyzik R, Goodman S, Chen X, et al.. (2013) Suppression of native defense mechanisms, SIRT1 and PPARgamma, by dietary glycoxidants precedes disease in adult humans; relevance to lifestyle-engendered chronic diseases. Amino Acids. 10.1007/s00726-013-1502-4 PMC3795943 23636469

[27] Uribarri J, Cai W, Ramdas M, Goodman S, Pyzik R, et al (2011) Restriction of advanced glycation end products improves insulin resistance in human type 2 diabetes: potential role of AGER1 and SIRT1. Diabetes Care 34: 1610–1616. 21709297 10.2337/dc11-0091 PMC3120204

[28] Liu TF, Vachharajani VT, Yoza BK, McCall CE (2012) NAD+-dependent sirtuin 1 and 6 proteins coordinate a switch from glucose to fatty acid oxidation during the acute inflammatory response. J Biol Chem 287: 25758–25769. 22700961 10.1074/jbc.M112.362343 PMC3406663

[29] Busch F, Mobasheri A, Shayan P, Stahlmann R, Shakibaei M (2012) Sirt-1 is required for the inhibition of apoptosis and inflammatory responses in human tenocytes. J Biol Chem 287: 25770–25781. 22689577 10.1074/jbc.M112.355420 PMC3406664

[30] Van de Wiele C, Maes A, Brugman E, D'Asseler Y, De Spiegeleer B, et al (2012) SIRT of liver metastases: physiological and pathophysiological considerations. Eur J Nucl Med Mol Imaging 39: 1646–1655. 22801733 10.1007/s00259-012-2189-6

[31] He W, Wang Y, Zhang MZ, You L, Davis LS, et al (2010) Sirt1 activation protects the mouse renal medulla from oxidative injury. J Clin Invest 120: 1056–1068. 20335659 10.1172/JCI41563 PMC2846063

[32] Li H, Rajendran GK, Liu N, Ware C, Rubin BP, et al (2007) SirT1 modulates the estrogen-insulin-like growth factor-1 signaling for postnatal development of mammary gland in mice. Breast Cancer Res 9: R1. 17201918 10.1186/bcr1632 PMC1851382

[33] Wu Z, Liu MC, Liang M, Fu J (2012) Sirt1 protects against thrombomodulin down-regulation and lung coagulation after particulate matter exposure. Blood 119: 2422–2429. 22262770 10.1182/blood-2011-04-350413

[34] Wei Q, Dong G, Chen JK, Ramesh G, Dong Z (2013) Bax and Bak have critical roles in ischemic acute kidney injury in global and proximal tubule-specific knockout mouse models. Kidney Int 84: 138–148. 23466994 10.1038/ki.2013.68 PMC3686831

[35] Ranganathan PV, Jayakumar C, Mohamed R, Dong Z, Ramesh G (2013) Netrin-1 regulates the inflammatory response of neutrophils and macrophages, and suppresses ischemic acute kidney injury by inhibiting COX-2-mediated PGE2 production. Kidney Int 83: 1087–1098. 23447066 10.1038/ki.2012.423 PMC3672333

[36] Chen T, Guo ZP, Wang L, Qin S, Cao N, et al (2013) Paeoniflorin suppresses vascular damage and the expression of E-selectin and ICAM-1 in a mouse model of cutaneous Arthus reaction. Exp Dermatol 22: 453–457. 23800055 10.1111/exd.12174

[37] Ranganathan P, Jayakumar C, Ramesh G (2013) Proximal tubule-specific overexpression of netrin-1 suppresses acute kidney injury-induced interstitial fibrosis and glomerulosclerosis through suppression of IL-6/STAT3 signaling. Am J Physiol Renal Physiol 304: F1054–1065. 23408169 10.1152/ajprenal.00650.2012 PMC3625840

[38] Araujo M, Doi SQ, Palant CE, Nylen ES, Becker KL (2013) Procalcitonin induced cytotoxicity and apoptosis in mesangial cells: implications for septic renal injury. Inflamm Res. 10.1007/s00011-013-0646-8 23872926

[39] Heuer JG, Zhang T, Zhao J, Ding C, Cramer M, et al (2005) Adoptive transfer of in vitro-stimulated CD4+CD25+ regulatory T cells increases bacterial clearance and improves survival in polymicrobial sepsis. J Immunol 174: 7141–7146. 15905557 10.4049/jimmunol.174.11.7141

[40] Doi K, Hu X, Yuen PS, Leelahavanichkul A, Yasuda H, et al (2008) AP214, an analogue of alpha-melanocyte-stimulating hormone, ameliorates sepsis-induced acute kidney injury and mortality. Kidney Int 73: 1266–1274. 18354376 10.1038/ki.2008.97 PMC2398767

[41] Dear JW, Yasuda H, Hu X, Hieny S, Yuen PS, et al (2006) Sepsis-induced organ failure is mediated by different pathways in the kidney and liver: acute renal failure is dependent on MyD88 but not renal cell apoptosis. Kidney Int 69: 832–836. 16518342 10.1038/sj.ki.5000165 PMC2271059

[42] Zager RA, Johnson AC, Hanson SY, Lund S (2006) Acute nephrotoxic and obstructive injury primes the kidney to endotoxin-driven cytokine/chemokine production. Kidney Int 69: 1181–1188. 16395275 10.1038/sj.ki.5000022

[43] Cunningham PN, Dyanov HM, Park P, Wang J, Newell KA, et al (2002) Acute renal failure in endotoxemia is caused by TNF acting directly on TNF receptor-1 in kidney. J Immunol 168: 5817–5823. 12023385 10.4049/jimmunol.168.11.5817

[44] Gautier EL, Huby T, Saint-Charles F, Ouzilleau B, Chapman MJ, et al (2008) Enhanced dendritic cell survival attenuates lipopolysaccharide-induced immunosuppression and increases resistance to lethal endotoxic shock. J Immunol 180: 6941–6946. 18453615 10.4049/jimmunol.180.10.6941

[45] Chen S, Zuo X, Yang M, Lu H, Wang N, et al (2012) Severe multiple organ injury in HSF1 knockout mice induced by lipopolysaccharide is associated with an increase in neutrophil infiltration and surface expression of adhesion molecules. J Leukoc Biol 92: 851–857. 22753951 10.1189/jlb.0212060

[46] Winnik S, Stein S, Matter CM (2012) SIRT1 - an anti-inflammatory pathway at the crossroads between metabolic disease and atherosclerosis. Curr Vasc Pharmacol 10: 693–696. 23259556 10.2174/157016112803520756

[47] Hwang JW, Chung S, Sundar IK, Yao H, Arunachalam G, et al (2010) Cigarette smoke-induced autophagy is regulated by SIRT1-PARP-1-dependent mechanism: implication in pathogenesis of COPD. Arch Biochem Biophys 500: 203–209. 20493163 10.1016/j.abb.2010.05.013 PMC2904411

[48] Yin H, Hu M, Zhang R, Shen Z, Flatow L, et al (2012) MicroRNA-217 promotes ethanol-induced fat accumulation in hepatocytes by down-regulating SIRT1. J Biol Chem 287: 9817–9826. 22308024 10.1074/jbc.M111.333534 PMC3323059

[49] Singer M (2013) Biomarkers in sepsis. Curr Opin Pulm Med 19: 305–309. 23411577 10.1097/MCP.0b013e32835f1b49 PMC4222798

[50] Gentile LF, Cuenca AG, Efron PA, Ang D, Bihorac A, et al (2012) Persistent inflammation and immunosuppression: a common syndrome and new horizon for surgical intensive care. J Trauma Acute Care Surg 72: 1491–1501. 22695412 10.1097/TA.0b013e318256e000 PMC3705923

[51] Mortuza R, Chen S, Feng B, Sen S, Chakrabarti S (2013) High glucose induced alteration of SIRTs in endothelial cells causes rapid aging in a p300 and FOXO regulated pathway. PLoS One 8: e54514. 23342163 10.1371/journal.pone.0054514 PMC3546959

[52] Scott MJ, Godshall CJ, Cheadle WG (2002) Jaks, STATs, Cytokines, and Sepsis. Clin Diagn Lab Immunol 9: 1153–1159. 12414743 10.1128/CDLI.9.6.1153-1159.2002 PMC130124

[53] Fan H, Yang HC, You L, Wang YY, He WJ, et al (2013) The histone deacetylase, SIRT1, contributes to the resistance of young mice to ischemia/reperfusion-induced acute kidney injury. Kidney Int 83: 404–413. 23302720 10.1038/ki.2012.394

[54] Kolaczkowska E, Kubes P (2013) Neutrophil recruitment and function in health and inflammation. Nat Rev Immunol 13: 159–175. 23435331 10.1038/nri3399

[55] Kong S, McBurney MW, Fang D (2012) Sirtuin 1 in immune regulation and autoimmunity. Immunol Cell Biol 90: 6–13. 22105513 10.1038/icb.2011.102

[56] Brunet A, Sweeney LB, Sturgill JF, Chua KF, Greer PL, et al (2004) Stress-dependent regulation of FOXO transcription factors by the SIRT1 deacetylase. Science 303: 2011–2015. 14976264 10.1126/science.1094637

[57] Guarente L (2005) Calorie restriction and SIR2 genes—towards a mechanism. Mech Ageing Dev 126: 923–928. 15941577 10.1016/j.mad.2005.03.013

[58] Jung J, Ko SH, Yoo do Y, Lee JY, Kim YJ, et al (2012) 5,7-Dihydroxy-3,4,6-trimethoxyflavone inhibits intercellular adhesion molecule 1 and vascular cell adhesion molecule 1 via the Akt and nuclear factor-kappaB-dependent pathway, leading to suppression of adhesion of monocytes and eosinophils to bronchial epithelial cells. Immunology 137: 98–113. 22862554 10.1111/j.1365-2567.2012.03618.x PMC3449251

[59] Yeung F, Hoberg JE, Ramsey CS, Keller MD, Jones DR, et al (2004) Modulation of NF-kappaB-dependent transcription and cell survival by the SIRT1 deacetylase. EMBO J 23: 2369–2380. 15152190 10.1038/sj.emboj.7600244 PMC423286

